# Prevalence of stigma towards mental illness among Portuguese healthcare professionals: a descriptive and comparative study

**DOI:** 10.3389/fpsyt.2024.1425301

**Published:** 2024-08-01

**Authors:** Inês N. Torres, Helena P. Pereira, Maria Beatriz P. Moreira, Sílvia Marina, Miguel Ricou

**Affiliations:** ^1^ Faculty of Medicine, University of Porto, Porto, Portugal; ^2^ Center for Health Technology and Services Research, Faculty of Medicine, University of Porto, Porto, Portugal

**Keywords:** mental health, mental disorders, social stigma, health personnel, prevalence, comparative study

## Abstract

**Background:**

Stigmatising attitudes among healthcare professionals can hinder access to healthcare, making it important to address this issue. This study aimed to investigate the prevalence of stigma related to mental illness among Portuguese healthcare professionals and to compare the results among mental health professionals, General Practitioners (GPs) and other health professionals.

**Methods:**

An online cross-sectional observational study was conducted in Portugal using Google Forms® to collect data. The data collection process lasted five months, from September 2023 to January 2024. Participants were recruited from various professional associations and Health Centre Groups, through a purposive sampling. The study used the Portuguese version of the Opening Minds Stigma Scale for Healthcare Providers (OMS-HC) to measure stigma which assesses three dimensions: attitudes towards disclosure and help-seeking, attitudes towards people with mental illness, and attitudes towards social distance.

**Results:**

A total of 292 healthcare professionals participated in the study. In Portugal, healthcare professionals displayed low to moderate levels of stigma towards mental illness (M = 22.17, SD = 5.41). Mental health professionals demonstrated significantly lower levels of stigma (M=20.37, SD=5.37) compared to other healthcare professionals (M=24.15, SD=4.71), including GPs (M=23.97, SD=5.03). Additionally, having a close friend or relative with mental illness seemed to be related with lower levels of stigma for the dimension attitudes towards social distance (M=6.93, SD=2.50), compared to not having one (M=7.60, SD=2.56). On the other hand, a personal history of mental illness indicated higher levels of stigma for the dimension disclosure and help-seeking (M=8.95, SD=3.07), compared to having no history of mental illness (M=8.16, SD=2.67).

**Conclusion:**

This study indicates that Portuguese healthcare professionals have stigmatising attitudes towards mental illness, although at low to moderate levels. Training and frequent interaction with people with mental illness seem to be associated with lower levels of stigma. Personal experience of mental illness seems to follow the opposite path regarding disclosure and seeking help. Thus, further research is necessary to evaluate the effectiveness of anti-stigma measures and deepen the study of the concept of self-stigma in healthcare professionals.

## Background

Stigma is a phenomenon that includes labelling, stereotyping, isolation, status loss, and discrimination. Asymmetric relationships can exacerbate these components (1). Stigma can be categorised in three main parts: stereotypes, prejudice, and discrimination. Stereotypes are biased beliefs or thoughts about an individual based on their categorisation within a certain group. Prejudice refers to the personal emotions that arise from preconceived beliefs, while discrimination refers to behaviours that result from prejudice (2). These concepts are interrelated.

Stigmatising actions, whether conscious or unconscious, are directed towards individuals perceived as different due to their gender, race, sexual practices, illness, or other conditions. Such actions can significantly impact both public and individual health, leading to emotional and stress disorders, delays in seeking appropriate healthcare, and premature termination of health treatments (3, 4).

Mental health stigma affects not only specific groups but also healthcare professionals (5). This is a significant issue as healthcare professionals are expected to provide assistance and demonstrate a high level of understanding of these issues. Stigma among healthcare professionals can result in lower rates of help-seeking among individuals with mental illness, potentially leading to higher levels of morbidity and mortality from organic diseases (6). Furthermore, research indicates that stigma can exacerbate mental illness (7, 8), perpetuating a harmful cycle that must be disrupted.

Various authors have proposed reasons for the stigma that healthcare professionals hold towards mental illness (9). This could be attributed to feelings of helplessness in addressing mental health issues, stemming from a perceived lack of ability to assist these individuals (10) which can reinforce their negative attitudes (11). Additionally. the lack of training, preparation, and adequate support to deal with mental illness has been well documented as a contributing factor (12). The presence of stigma among healthcare professionals can be attributed to the fact that they are also citizens and part of the general population that experiences stigmatisation.

However, it seems that even mental health professionals continue to experience stigma, contradicting some of the arguments presented (5, 13). Studies in the literature have compared stigma among health professionals who have received specific mental health training and those who have not (6). This is an important comparison to determine whether mental health training can mitigate the development of stigma in healthcare professionals.

A study conducted in Russia found that psychiatrists and other health professionals tended to socially distance themselves from people with mental illness, despite having greater knowledge and professional experience in dealing with these patients (14). Similarly, research has shown that healthcare professionals with mental health training do not differ significantly from other health professionals in their decision-making regarding people with mental illness (6).

In contrast, a study conducted in Sweden found that individuals with higher levels of mental health literacy exhibited lower levels of stigma and social distance towards patients with depression (15). Similarly, a study conducted in Portugal supported these findings, revealing that psychiatrists exhibited lower levels of stigma than medical students and non-psychiatrist doctors (16).

Some literature suggests that personal experience of mental illness may be related to lower levels of stigma. Professionals with personal experience of mental illness report less stigma and social distance towards people with mental illness (15). Additionally, the mental health history of relatives or close friends also appears to be relevant, as studies have shown that individuals in this situation report lower levels of stigma (15, 16).

Additional research is necessary to evaluate the effects of stigma in various occupational groups. Several scales are currently available to measure stigma. The Opening Minds Stigma Scale for Healthcare Providers (OMS-HC) (17) is the only validated scale to assess stigma related to mental illness among healthcare professionals in several countries (18), including Portugal, in a 12-item version (19).

This study aims to investigate the prevalence of mental illness-related stigma among Portuguese healthcare professionals. It also aims to compare stigma levels between different groups of professionals, such as mental health professionals (psychiatrists, psychologists, mental health nurses, and occupational therapists specializing in mental health), General Practitioners (GPs), and other healthcare professionals. Likewise, this study seeks to understand whether differences in stigma exist among these groups. Finally, we intend to study the difference in levels of stigma between having a personal experience of mental illness and not, as well as having close contact with family members or friends who have suffered from mental illness and not having.

## Material and methods

### Study design

A quantitative, observational cross-sectional study was conducted to investigate the stigma of mental illness among healthcare professionals in Portugal.

### Participants

The study was conducted with 292 healthcare professionals from Portugal who were recruited through a purposive sampling. The sample size was calculated based on data from the most recent report of the National Statistics Institute of Portugal, which estimates that there are approximately 100,000 healthcare professionals in the country (20, 21). To ensure a 90% confidence interval and a 5% margin of error, a minimum sample size of 272 participants was required for this population (20).

Of the participants, 241 (82.1%) were female and 51 (17.5%) were male. The mean age of the participants was 42.77 years, ranging from 23 to 75 years. The study included a variety of professional groups, with 33 (11.3%) being GPs and 37.0% being other health professionals, such as internal medicine residents, physiotherapists, pharmacists, speech and language therapists, general nurses, and nutritionists. The sample consisted of mental health professionals, with psychologists accounting for 41.0% (N=62), mental health nurses 30.6% (N=46), psychiatrists 23.2% (N=35), and occupational therapists specialising in mental health 5.2% (N=8). On average, participants had 17.82 years of professional experience.

Furthermore, 34.9% (N=102) of participants reported a history of mental illness for which they had sought professional help. Additionally, 62.3% (N=182) had a direct relative or close friend with a mental illness with whom they regularly interacted (refer to **Table 1**).

**Table 1:** Sociodemographic data of the study participants.

Sociodemographic data	n=292
**Age**	mean **=** 42.77 years; SD = 10.26 min= 23; max= 75
Gender	
Female	241 (82.10%)
Male	51 (17.50%)
Professional groups	
General Practitioners	33 (11.30%)
Psychiatrists	35 (12.00%)
Psychologists	62 (21.20%)
Mental Health Nurses	46 (15.80%)
Occupational Therapists specializing in Mental Health	8 (2.70%)
Other healthcare professionals	108 (37.00%)
**Years of professional experience**	mean = 17.82 years; SD =10.26 min=0; max=44
Previous history of mental illness	
Yes	102 (34.90%)
No	109 (65.10%)
Direct relative or close friend with mental illness	
Yes	182 (62.30%)
No	110 (37.70%)

n=292.

### Instruments

This study used the Portuguese version of the Opening Minds Stigma Scale for Healthcare Providers (OMS-HC) to evaluate the attitude of healthcare professionals towards mental illness (19). The scale comprises 12 items and three subscales: factor one, 'Attitudes of healthcare professionals towards disclosure and seeking help' (including items 1, 3, 4 and 5); factor two, 'Attitudes of healthcare professionals towards individuals with mental illness' (including items 8, 9, 10 and 12); and factor three, 'Attitudes of healthcare professionals towards social distance' (including items 2, 6, 7 and 11). Each item is quantitatively scored using a Likert scale with the following options: 'strongly disagree', 'disagree', 'neither agree nor disagree', 'agree' and 'strongly agree'. Items 2, 6, 7 and 11 are scored in reverse order. The total possible scores for the 12-item OMS-HC range from 12 to 60, with lower scores indicating less stigmatising attitudes and higher scores indicating more stigmatising attitudes. Subscale scores range from 4 to 20. The Cronbach's alpha for the original version of the OMS-HC 15 scale was 0.79, while the Cronbach's alpha for the 12-item Portuguese version was 0.71. In Portuguese version, the Cronbach’s alpha for factor 1 was 0.67, factor 2 was 0.62 and 0.60 for factor 3.

A socio-demographic questionnaire was also administered, which included variables such as age, gender, field of work, years of work experience, personal history of mental illness, and presence of a close relative with a mental illness. Additionally, self-perception of stigma was assessed using a 10-point Likert scale, specifically designed by the authors for this study, ranging from 0 (no stigma) to 10 (very high stigma). Awareness of stigma was also evaluated by answering a question designed by the authors.

### Procedures

Following approval from the Ethics Committee of the São João Hospital and University Centre, we requested approval and collaboration from the Health Centre Groups that comprise the Northern Regional Health Administration of Portugal. Once we received approval from all the Health Centre Groups, we submitted the project to the Ethics Committee of the Northern Regional Health Administration of Portugal for study approval. Healthcare organisations, such as professional orders, associations, societies, and some hospitals, were invited to collaborate via email.

Once accepted, the researchers sent the Google Forms® link to the collaborating institutions, who were then responsible for sharing the link with their professionals and inviting them to participate in the study. The data collection process lasted five months, from September 2023 to January 2024, and did not require authentication to ensure participant anonymity. Informed consent was obtained, and all participants agreed to the use of their data for research purposes. This step was necessary to complete the questionnaire. Before collecting the data, a pilot study was conducted with 14 participants to ensure that the questionnaire was understandable and suitable for the study population.

### Statistical data analysis

The Statistical Package for the Social Sciences (SPSS) 27.0 for Windows was used to analyse the variables. Descriptive analyses were performed to calculate the mean and standard deviation of the variable to assess the prevalence of stigma towards mental illness among healthcare professionals. The total score of the OMS-HC scale was calculated, with items 2, 6, 7 and 11 reversed beforehand. The demographic variables of the study sample were analysed descriptively using percentages, means, and standard deviations.

To compare stigma levels between different professional groups, a one-way ANOVA analysis was performed on mental health professionals, GPs, and other health professionals. Parametric statistics were chosen because the sample distribution was normal (|Sk|<3 and |Ku|<10), and the variances were homogeneous according to Levene's test (p>0.05). The significance level for the p-value was set at less than 0.05. The effect size was measured using Eta squared (η²). To better understand group differences, the *post-hoc* Tukey HSD test was employed.

Stigma scores were compared based on personal history and having a relative or close friend with mental illness using independent samples t-tests. Levene's test confirmed the assumption of homogeneity of variances (p>0.05). Statistical significance was determined using one-tailed p-values with a threshold of <0.05. Effect size was measured using Cohen's d value.

## Results

A total of 292 healthcare professionals participated in the study. The mean prevalence of stigma towards mental illness among Portuguese healthcare professionals was 22.17 (SD=5.41), with stigma scores ranging from 12 to 44. The prevalence of stigma for each factor of the OMS-HC scale was assessed. Factor one, which pertains to attitudes towards disclosure and seeking help, had a mean score of 8.43 (SD=2.84). Factor two, which pertains to attitudes towards people with mental illness, had a mean score of 6.55 (SD=2.48). Factor three, which pertains to attitudes of health professionals towards social distance, had a mean score of 7.18 (SD=2.54). Stigma scores for all subscales ranged from 4 to 20. **Table 2** shows the means and standard deviations for the total scale and each factor.

**Table 2:** Mean and standard deviation of the total prevalence of stigma and the prevalence of stigma for each item of the scale.

	Mean	SD	*Skewness*	*Kurtosis*
**Total OMS-HC scale**	22.17	5.41	0.37	-0.02
**Factor “Attitudes towards disclosure and seeking help”**	8.43	2.84	0.61	0.43
**Factor “Attitudes towards people with mental illness”**	6.55	2.48	1.14	2.29
**Factor “Healthcare professionals' attitudes towards social distance”**	7.18	2.54	0.75	1.13

n=292.

The one-way ANOVA performed indicated statistically significant differences among the three professional groups in terms of the total score on the OMS-HC scale (F(2.509) = 19.63, p < 0.001, η² = 0.12). *Post-hoc* analysis using the Tukey HSD test revealed significant differences between mental health professionals and the other groups, with p-values < 0.001. No significant differences were found between the group of GPs and other health professionals (p = 0.983). Mental health professionals exhibited lower stigma scores (M=20.37, SD=5.37) compared to GPs (M=23.97, SD=5.03) and other health professionals (M=24.15, SD=4.71). Statistically significant differences were also observed for factor one [F (2.189) = 27.68, p <0.001, η² = 0.16] and factor three [F(2.43) = 6.90, p = 0.001, η² = 0.05], but not for factor two [F(2.8) = 1.24, p = 0.292, η² = 0.008]. As with the total scale, significant differences were found for mental health professionals compared with GPs and other health professionals for factors one and three (refer to **Table 3**).

**Table 3:** One-way ANOVA results comparing the three groups of health professionals in terms of the prevalence of stigma towards mental illness.

	Mental health professionals		General Practitioners		Other healthcare professionals		ANOVA			
	M	SD	M	SD	M	SD	F ratio	df	*p*	η2
**Total OMS-HC scale**	20.37	5.37	23.97	5.03	24.15	4.71	19.63	2	<0.001	0.12
**Factor “Attitudes towards disclosure and seeking help”**	7.34	2.55	9.76	2.50	9.56	2.73	27.68	2	<0.001	0.16
**Factor “Attitudes towards people with mental illness”**	6.37	2.67	6.42	2.08	6.85	2.29	1.24	2	0.292	0.05
**Factor “Healthcare professionals' attitudes towards social distance”**	6.66	2.54	7.79	2.29	7.73	2.47	6.90	2	0.001	0.01

n=292.

When comparing the difference of having or not having a personal history of mental illness between the groups, statistically significant differences were found only for factor one 'attitudes towards disclosure and seeking help' (t (290) = -2.29, p = 0.011, d = -0.28, 95% CI [-1.47, -0.11]). In this case, stigma scores were higher for those with a history of mental illness (M=8.95, SD=3.07) compared to those without a history of mental illness (M=8.16, SD=2.67) (see **Table 4**).

**Table 4:** Results of independent samples t-tests to compare the prevalence of mental illness stigma between groups.

	Personal history of mental illness		No personal history of mental illness		*t*(290)	*p*	Cohen's d
	M	SD	M	SD			
**Total OMS-HC scale**	22.27	5.73	22.12	5.25	-0.23	0.409	-0.03
**Factor “Attitudes towards disclosure and seeking help”**	8.95	3.07	8.16	2.67	-2.29	0.011	-0.28
**Factor “Attitudes towards people with mental illness”**	6,26	2.38	6.71	2.53	1.47	0.072	0.18
**Factor “Healthcare professionals' attitudes towards social distance”**	7.06	2.75	7.25	2.42	0.62	0.267	0.08
	Relative or close friend with a mental illness		No relative or close friend with a mental illness		*t*(290)	*p*	Cohen's d
	M	SD	M	SD			
**Total OMS-HC scale**	21.74	5.45	22.89	5.28	1.77	0.039	0.21
**Factor “Attitudes towards disclosure and seeking help”**	8.39	2.88	8.51	2.79	0.35	0.365	0.42
**Factor “Attitudes towards people with mental illness”**	6.42	2.41	6.78	2.59	1.22	0.116	0.15
**Factor “Healthcare professionals' attitudes towards social distance”**	6.93	2.50	7.60	2.56	2.19	0.015	0.26

n=292.

Finally, significant differences were found for the total OMS-HC scale (t(290) = 1.77, p = 0.039, d = 0.21, 95% CI [-0.13, 2.43]) and for factor three (t(290) = 2.19, p = 0.015, d = 0.26, 95% CI [0.66, 1.27]) when comparing the differences between having or not having a relative or close friend with a history of mental illness. **Table 4** shows that the group with a relative or friend diagnosed with a mental illness exhibits lower levels of stigma than the group without any close acquaintances with a mental illness, for both the total scale and factor 3 (social distance).

The self-report of stigma after completing the questionnaire obtained an average score of 2.03 (SD=0.64). Furthermore, 55.5% of participants reported experiencing little or no stigma towards mental illness (responses ranging from 0 to 1 out of 10). When asked whether completing the scale had increased their awareness of their own stigma, 51.4% of participants reported that it had not.

## Discussion

Given that the OMS-HC scale ranges from 12 to 60, with 12 representing a complete absence of stigma and 60 representing a maximum level of stigma, an average score of 22 for the total scale, as obtained in this study, may indicate a level of stigma below what a median score could indicate. So, the stigma of Portuguese healthcare professionals towards mental illness in this study can be considered low to moderate. A similar OMS-HC scale was used in southern India to investigate mental health stigma among doctors of different specialties, and the results showed a low to moderate prevalence of stigma, consistent with our findings (22). In other countries, Hungarian and Canadian psychiatrists obtained an average stigma score of 29 points and 23 points respectively, for the full OMS-HC scale (23, 24). A Spanish study also found a low to moderate prevalence (25). A study in the USA attempted to create a new scale to measure stigma among health professionals based on the previous experiences of people with mental illness and their families. The authors found an average prevalence of stigma, which they considered to be low to moderate (26). The prevalence of stigma regarding mental illness among Portuguese health professionals appears to be consistent with that in other countries, and in some cases lower than that obtained in similar studies and even lower than those obtained in studies using the OMS-HC scale.

When comparing mental health professionals with other health professionals and GPs, significant differences were found between the groups. Mental health professionals exhibited less stigma towards mental illness than all other health professionals. However, GPs did not show less stigma than other health professionals. As GPs are the gateway to the National Health Service (NHS) and are often have contact with people suffering from mental illness in their professional practice, specifically emotional disorders such as anxiety and depressive disorders, we expected them to show lower levels of stigma. However, specific training in mental health, combined with working exclusively in this area, seems to be associated with lower levels of stigma than working non-exclusively with people with mental illnesses.

There is conflicting information in the international literature on these findings. A study (6) found no significant differences between mental health professionals and GPs regarding stigmatising attitudes and expectations of treatment adherence among patients. Other studies suggest that mental health professionals may have similar or higher levels of stigma than professionals from other fields (5, 13). Additionally, other study found that both psychiatrists and other health professionals are likely to exhibit similar levels of social distancing from people with mental illness (14). Likewise, a study conducted in Greece concluded that a high level of contact with patients suffering from mental illness is not necessarily associated with lower levels of stigma (27). On the other hand, a study conducted in Portugal found that psychiatrists had lower levels of stigma compared to students and doctors from other specialities. It did not specify whether the other health professionals were GPs (16). Moreover, a study involving medical trainees from various specialties in Iran found that those specializing in psychiatry exhibited less stigma towards individuals with mental illness (28).

The marked differences may be related to the division of the OMS-HC into three factors. The results show that all professional groups surveyed have similar levels of stigma regarding 'attitudes towards the person with mental illness' (factor 2), which is the factor with a relatively low level of stigma. In contrast, the factor that shows a higher level of stigma is 'attitudes towards disclosure and seeking help' (factor 1), followed by 'attitudes towards social distance' (factor 3). These two factors show the greatest differences between mental health professionals and others. A possible explanation could be related to social desirability. It is more difficult to accept discriminatory behaviour towards people with mental illness than to accept having difficulties with mental illness itself. In fact, it is more difficult to consider as stigma the fact that a person does not want to reveal their mental health problem or has difficulties in seeking help than to have negative attitudes towards people with mental illness, where stigma is explicit (28). Therefore, if only factor 2 were considered in this study, there would be no differences between the groups and stigma would be lower among healthcare professionals.

Having a close friend or relative with a mental illness seems to be related with lower levels of stigma, as the group of participants who identified themselves as such showed lower levels of stigma. The results suggest that the difference between the groups is primarily due to factor 3, social distance, which relates to avoiding contact with people with mental illness. It is logical to assume that familiarity with mental illness, through personal relationships, would lead to a decrease in avoidance of others with mental illness. A study conducted in Portugal supports these findings, concluding that doctors with a relative diagnosed with mental illness had less stigmatising attitudes (16). Similarly, a study conducted in Sweden shows that having a relative with mental illness is associated with less stigma and social distance from healthcare professionals towards patients with depression (15).

On the other hand, the results of this study indicate that the group of participants with a personal history of mental illness did not show significant differences in stigma levels compared to the others, except for factor 1, disclosure and seeking help. At this level, the group with a personal history of mental illness showed higher levels of stigma than participants without such a background. This result may indicate the real difficulties people feel when faced with a mental health problem, which could be characterised as self-stigma. This could increase difficulties in seeking help and may be due to a sense of misunderstanding, whether true or false, by others about their problems. It is uncertain whether individuals construct their own difficulties in accepting and understanding their symptoms, or if these difficulties result from the attitudes of others, or both. However, it is important to break this self-perpetuating cycle that exacerbates the problems associated with mental illness. This can be achieved by reducing the likelihood of seeking help or by increasing social isolation.

The study conducted in Hungary yielded mixed results regarding the disclosure and help-seeking factor. Approximately 50% of Hungarian psychiatrists expressed reluctance to seek help for their own mental illness and were unwilling to share their diagnosis with colleagues (23). This finding is consistent with the notion that mental illness may be perceived in the professional environment as indicating less competence, greater danger, and unpredictability. This perception may lead to the belief that a professional with a mental illness should not work with people who also have a mental illness (29). Additionally, self-stigma is common among people with mental illness (30). It may cause individuals to fear being perceived as incapable or untrustworthy, and to be labelled based on their diagnosis (31).

A recent study conducted in Portugal measured mental health stigma among healthcare professionals and found no significant differences in personal experience of mental illness (32). Similarly, a validation study of the OMS-HC scale in Italy also found no differences in stigma related to personal experience of mental illness among students (33). Conversely, a study performed in Sweden found that individuals with a personal history of mental illness experienced lower levels of stigma and social distancing towards patients with depression (15). Other studies suggest that greater familiarity with mental illness, such as being comfortable seeking mental health care for oneself, may lead to less stigma. This reinforces the need to seek help as a way of deconstructing stigma (34).

So, it appears that individuals with a history of mental illness in their close circle have lower levels of stigma, particularly related to factor 3 (social distance). However, no reduction in stigma was found for individuals with a personal history of mental illness. In fact, there was an increase in stigma related to factor 1, disclosure and seeking help.

Overall, the study participants reported a low level of stigma towards mental illness. Only 48.6% believed that completing the questionnaire had contributed to raising awareness of this issue. Differences found at the factor level may indicate a lack of awareness of the existing stigma, which is not very high but still present. The participants did not have access to their own results. This could be positive if completing the questionnaire was linked to a measure to combat stigma. Studies have shown that taking part in anti-stigma interventions can increase the awareness of healthcare professionals of the stigma they themselves feel towards mental illness and the unintentional attitudes that reveal it (35–38).

### Limitations

This study may have limitations regarding the extrapolation of conclusions to the Portuguese healthcare population. Firstly, this is a non-random sampling, which is known to limit the representativeness of the population being studied. Also, the response rate to this type of study is typically low. Additionally, the population most motivated to respond to the questionnaire is usually the one most interested in the topic, which may introduce some bias. Furthermore, it is important to note that the sample size of GPs was relatively small. Therefore, any comparisons made between professional groups should be interpreted with caution, as the group of GPs is smaller than the others.

Another limitation of the study is that this way of measuring stigma is based on a self-prediction model of stigmatising acts rather than the stigmatising acts themselves. The theoretical model only assesses intentions, which may differ from reality. Healthcare professionals may exhibit behaviours that differ from what they admit to in the questionnaire. Further research is required to evaluate stigma in alternative ways, particularly to ascertain the perception of stigma by individuals with mental illness towards their healthcare providers. Moreover, this is a cross-sectional study, it only provides a static evaluation of stigma over time and not a continuous assessment, which precludes the evaluation of changes in stigma over time.

## Conclusion

This research indicates that Portuguese health professionals hold stigmatising attitudes towards mental illness, although at a low to moderate level. However, training and frequent intervention with individuals with this condition, as is the case with mental health professionals, appear to be related to lower levels of stigma. Nonetheless, it is important to create anti-stigma programmes, particularly for GPs (who are typically the first point of contact for individuals with mental illness and refer them to the NHS), to reduce the likelihood of alienating these individuals from mental health care.

Further research is required to continuously assess stigma over time and evaluate the effectiveness of anti-stigma interventions, such as mental health training, which can increase mental health literacy and promote closer contact with patients. The OMS-HC scale can be useful for pre-intervention evaluations of anti-stigma interventions and for post-intervention evaluations to study their effectiveness. Moreover, future studies are necessary in order to understand the influence of gender, age, culture, years of professional experience and other variables in stigma levels. It could also be studied the relative stigma related to each specific mental disorder and how the different components of stigma relate to each other.

Contact with mental illness appears to protect against the development of stigmatising attitudes, particularly social distancing. Conversely, personal experience with a history of mental illness appears to increase stigma, particularly in the expectation of acceptance from others, including health professionals. It is important to study self-stigma to prevent it from influencing care.

## Data availability statement

The original contributions presented in the study are included in the article/supplementary material. Further inquiries can be directed to the corresponding author.

## Ethics statement

The studies involving humans were approved by Comissão de Ética do Centro hospitalar e universitário São João/ Faculdade de Medicina da Universidade do Porto. The studies were conducted in accordance with the local legislation and institutional requirements. The participants provided their written informed consent to participate in this study.

## Author contributions

INT: Writing – original draft, Writing – review & editing. HP: Writing – original draft, Writing – review & editing. MBPM: Writing – review & editing. SM: Writing – review & editing. MR: Writing – original draft, Writing – review & editing.

## References

[1] Link BG Phelan JC. Conceptualizing stigma. Annu Rev Sociology. (2001) 27:363–85. doi: 10.1146/annurev.soc.27.1.363

[2] Fiske ST. Prejudice, discrimination, and stereotyping. In: Biswas-Diener R Diener E, editors. Noba textbook series: Psychology. Champaign, IL: DEF Publishers (2024). Available from: https://noba.to/jfkx7nrd.

[3] Link BG Phelan JC. Stigma and its public health implications. Lancet. (2006) 367:528–9. doi: 10.1016/S0140-6736(06)68184-1 16473129

[4] Weiss MG Ramakrishna J Somma D. Health-related stigma: Rethinking concepts and interventions 1. Psychology Health Med. (2006) 11:277–87. doi: 10.1080/13548500600595053 17130065

[5] Schulze B. Stigma and mental health professionals: a review of the evidence on an intricate relationship. Int Rev Psychiatry. (2007) 19:137–55. doi: 10.1080/09540260701278929 17464792

[6] Corrigan PW Mittal D Reaves CM Haynes TF Han X Morris S. Mental health stigma and primary health care decisions. Psychiatry Res. (2014) 218:35–8. doi: 10.1016/j.psychres.2014.04.028 PMC4363991 24774076

[7] Rüsch N Angermeyer MC Corrigan PW. Mental illness stigma: Concepts, consequences, and initiatives to reduce stigma. Eur Psychiatry. (2005) 20:529–39. doi: 10.1016/j.eurpsy.2005.04.004 16171984

[8] Hinshaw SP Stier A. Stigma as related to mental disorders. Annu Rev Clin Psychol. (2008) 4:367–93. doi: 10.1146/annurev.clinpsy.4.022007.141245 17716044

[9] Batista L. Os profissionais de saúde eo estigma da doença mental. Universidade do Porto. Instituto de Ciências Biomédicas de Abel Salazar, Porto (2013).

[10] Cohen NL. Stigma is in the eye of the beholder: a hospital outreach program for treating homeless mentally ill people. Bull Menninger Clin. (1990) 54:255–8. 2364212

[11] Knaak S Modgill G Patten SB. Key ingredients of anti-stigma programs for health care providers: a data synthesis of evaluative studies. Can J Psychiatry. (2014) 59:19–26. doi: 10.1177/070674371405901S06 PMC4213748 25565698

[12] Minkoff K. Resistance of mental health professionals to working with the chronic mentally ill. New Dir Ment Health Serv. (1987) 1987:3–20. doi: 10.1002/yd.23319873303 3574266

[13] Lauber C Nordt C Braunschweig C Rössler W. Do mental health professionals stigmatize their patients? Acta Psychiatr Scand Suppl. (2006) 429:51–9. doi: 10.1111/j.1600-0447.2005.00718.x 16445483

[14] Yashikhina A Romanov D Strelnik S Gradinar A Markina E Smirnova D. Non-psychiatrist healthcare professionals' attitudes toward patients with mental disorders: Lower scores in social distance as a fragile facet of public stigma against depression [Conference paper]. Psychiatr Danub. (2023) 34(Suppl 8):238–245. 36170737

[15] Svensson B Hansson L. How mental health literacy and experience of mental illness relate to stigmatizing attitudes and social distance towards people with depression or psychosis: A cross-sectional study. Nordic J Psychiatry. (2016) 70:309–13. doi: 10.3109/08039488.2015.1109140 26643359

[16] Oliveira AM MaChado D Fonseca JB Palha F Silva Moreira P Sousa N. Stigmatizing attitudes toward patients with psychiatric disorders among medical students and professionals. Front Psychiatry. (2020) 11:326. doi: 10.3389/fpsyt.2020.00326 32425827 PMC7207477

[17] Modgill G Patten SB Knaak S Kassam A Szeto AC. Opening minds stigma scale for health care providers (OMS-HC): examination of psychometric properties and responsiveness. BMC Psychiatry. (2014) 14:1–10. doi: 10.1186/1471-244X-14-120 PMC4024210 24758158

[18] Őri D Szocsics P Molnár T Bankovska Motlova L Kazakova O Mörkl S. Psychometric properties of the Opening Minds Stigma Scale for Health Care Providers in 32 European countries - A bifactor ESEM representation. Front Public Health. (2023) 11:1168929. doi: 10.3389/fpubh.2023.1168929 37361150 PMC10285467

[19] Moreira MBP Pereira HP Torres IN Marina S Ricou M. The stigma towards mental illness: Portuguese validation of the Opening Minds Stigma Scale for Healthcare Providers (OMS-HC). Front Psychol (2024) 15:1359483. doi: 10.3389/fpsyg.2024.1359483 PMC10955081 38515965

[20] Charan J Biswas T. How to calculate sample size for different study designs in medical research? Indian J Psychol Med. (2013) 35(2):121–6. doi: 10.4103/0253-7176.116232 PMC3775042 24049221

[21] Instituto Nacional de Estatística [National Institute of Statistics]. Estatísticas da Saúde: 2020 [Health Statistics: 2020]. Lisboa: INE (2020). Disponível em: https://www.ine.pt/xurl/pub/436989156.

[22] Munisami T Namasivayam RK Annamalai A. Mental-illness-related stigma in health care in South India: mixed-methods study. Indian J Psychol Med. (2021) 43:58–64. doi: 10.1177/0253717620932244 34349308 PMC8295582

[23] Őri D Szocsics P Molnár T Ralovich FV Huszár Z Bene Á. Stigma towards mental illness and help-seeking behaviors among adult and child psychiatrists in Hungary: A cross-sectional study. PloS One. (2022) 17:e0269802. doi: 10.1371/journal.pone.0269802 35687584 PMC9187077

[24] Dabby L Tranulis C Kirmayer LJ. Explicit and implicit attitudes of Canadian psychiatrists toward people with mental illness. Can J Psychiatry. (2015) 60:451–9. doi: 10.1177/070674371506001006 PMC4679121 26720192

[25] Valverde-Bolivar E Garcia-Arenas JJ Lopez Pelegrin I Perez-Gomez L Munoz-Lopez M Simonelli-Munoz AJ. The stigma of mental health professionals towards users with a mental disorder. Actas Esp Psiquiatr. (2022) 50(4):178–86. PMC10803850 35867484

[26] Charles JLK Bentley KJ. Measuring mental health provider-based stigma: development and initial psychometric testing of a self-assessment instrument. Community Ment Health J. (2018) 54:33–48. doi: 10.1007/s10597-017-0137-4 28378302

[27] Porfyri GN Athanasiadou M Siokas V Giannoglou S Skarpari S Kikis M. Mental health-related stigma discrimination and prejudices among Greek healthcare professionals. Front Psychiatry. (2022) 13:1027304. doi: 10.3389/fpsyt.2022.1027304 36532175 PMC9757138

[28] Movahedi S Shariat SV Shalbafan M. Attitude of Iranian medical specialty trainees toward providing health care services to patients with mental disorders. Front Psychiatry. (2022) 13:961538. doi: 10.3389/fpsyt.2022.961538 35966498 PMC9366058

[29] Krupa T Kirsh B Cockburn L Gewurtz R. Understanding the stigma of mental illness in employment. Work. (2009) 33:413–25. doi: 10.3233/WOR-2009-0890 19923664

[30] Dubreucq J Plasse J Franck N. Self-stigma in serious mental illness: A systematic review of frequency, correlates, and consequences. Schizophr Bull. (2021) 47:1261–87. doi: 10.1093/schbul/sbaa181 PMC8563656 33459793

[31] Cohen D Winstanley S Greene G. Understanding doctors’ attitudes towards self-disclosure of mental ill health. Occup Med. (2016) 66:383–9. doi: 10.1093/occmed/kqw024 PMC4913366 27030052

[32] Rodrigues D Adão C Sequeira A. Mental Illness Stigma among professionals at a Portuguese Medical Center. Eur Psychiatry. (2023) 66:S353–S. doi: 10.1192/j.eurpsy.2023.769

[33] Destrebecq A Ferrara P Frattini L Pittella F Rossano G Striano G. The Italian version of the opening minds stigma scale for healthcare providers: validation and study on a sample of bachelor students. Community Ment Health J. (2018) 54:66–72. doi: 10.1007/s10597-017-0149-0 28647819

[34] Corrigan PW Edwards AB Green A Diwan SL Penn DL. Prejudice, social distance, and familiarity with mental illness. Schizophr Bull. (2001) 27:219–25. doi: 10.1093/oxfordjournals.schbul.a006868 11354589

[35] Knaak S Patten S. A grounded theory model for reducing stigma in health professionals in Canada. Acta Psychiatrica Scandinavica. (2016) 134:53–62. doi: 10.1111/acps.12612 27426646

[36] Sukhera J Chahine S. Reducing mental illness stigma through unconscious bias-informed education. MedEdPublish. (2016) 5:44. doi: 10.12688/mep

[37] Horsfall J Cleary M Hunt GE. Stigma in mental health: Clients and professionals. Issues Ment Health Nursing. (2010) 31:450–5. doi: 10.3109/01612840903537167 20521914

[38] Michaels PJ Corrigan PW. Measuring mental illness stigma with diminished social desirability effects. J Ment Health. (2013) 22:218–26. doi: 10.3109/09638237.2012.734652 23323874

